# Can genomics deliver climate-change ready crops?

**DOI:** 10.1016/j.pbi.2018.03.007

**Published:** 2018-10

**Authors:** Rajeev K Varshney, Vikas K Singh, Arvind Kumar, Wayne Powell, Mark E Sorrells

**Affiliations:** 1 Center of Excellence in Genomics & Systems Biology, International Crops Research Institute for the Semi-Arid Tropics (ICRISAT), Patancheru 502324, India; 2 International Rice Research Institute (IRRI), IRRI South Asia Hub, ICRISAT, Patancheru 502324, India; 3 SRUC (Scotland's Rural College), Peter Wilson Building, West Mains Road, Edinburgh EH9 3JG, UK; 4 Department of Plant Breeding, 240 Emerson Hall, Cornell, Ithaca, NY 14853-1902, USA

## Abstract

Development of climate resilient crops with accelerating genetic gains in crops will require integration of different disciplines/technologies, to see the impact in the farmer's field. In this review, we summarize how we are utilizing our germplasm collections to identify superior alleles/haplotypes through NGS based sequencing approaches and how genomics-enabled technologies together with precise phenotyping are being used in crop breeding. Pre-breeding and genomics-assisted breeding approaches are contributing to the more efficient development of climate-resilient crops. It is anticipated that the integration of several disciplines/technologies will result in the delivery of climate change ready crops in less time.

## Highlights

• Sequencing and phenotyping of genebanks can provide superior alleles/haplotypes.
• NGS and precise phenotyping can be used for fast forward trait mapping.
• Pre-breeding is suggested to identify/introduce alleles for climate-change traits.
• Genomic selection and genome editing are the next generation breeding approaches.
• Integration of different disciplines is required to develop climate resilient crops.

**Current Opinion in Plant Biology** 2018, **45**:205–211

This review comes from a themed issue on **AGRI 2017**

Edited by **David Edwards**

For a complete overview see the Issue and the Editorial

Available online 21st April 2018

**https://doi.org/10.1016/j.pbi.2018.03.007**

1369-5266/© 2018 The Authors. Published by Elsevier Ltd. This is an open access article under the CC BY license (http://creativecommons.org/licenses/by/4.0/).

## Introduction

Based on recent reports and simulation studies it has been predicted that climate change is likely to have an adverse effect on the global yields of all major crops [1, 2]. Although crop improvement has made progress, the realized genetic gains in farmer's fields, especially in rainfed conditions has been very low [3]. Accelerating the rate of genetic gain to mitigate climate change to meet the target demands of food production, requires integration of multidisciplinary research platforms/disciplines [4, 5, 6••].

Genetic gain in research plots is directly proportional to first, the genetic variation (*σ*_*A*_), second, the intensity of selection (*i*) (i.e. the proportion of individuals not contributing to the next generation), and third, the selection accuracy (*r*) and inversely proportional to years per cycle (*y*) [7]. The breeder's equation originally conceived by Lush [8] provides a framework to guide, quantify and monitor interventions. Integration of modern genomics approaches for example next generation sequencing (NGS), high-throughput genotyping together with high throughput phenotyping (phenomics) and informatics and decision support tools can accelerate genetic gains over time [9^••^]. Selection intensity (*i*) can be increased if we can screen more plants per unit time or area. Advances in phenotyping (such as disease plots, artificial screening in labs/greenhouse, etc.) can accelerate the screening of a large number of plants for the trait of interest. Molecular markers can be used as proxies for phenotypic characteristics allowing selection to be performed on young plants and/or in early generations. Availability of high-throughput sequencing/genotyping platforms can allow assaying of thousands of plants in relatively short time. Selection accuracy (*r*) can be increased by using trait-linked markers that allow off-season selection in any location. For instance, in a year with adequate precipitation, selection of drought-tolerant plants is not possible in traditional breeding approaches. However, robust genomics-assisted breeding (GAB) approaches can be utilized in any season or any stage of plant growth [10^•^]. Genetic variance (*σ*_*A*_) can be increased by selecting those lines that have favorable but rare alleles for a trait(s) of interest. Years per cycle (*y*) can be reduced by growing more generations per year through rapid generation (3–6 crop seasons per year) advancement and/or speed breeding [11^••^], instead of 1 or 2 crop seasons per year. Speed breeding can be combined with selection on a single plant basis using visual selection or molecular markers.

**Figure 1:**
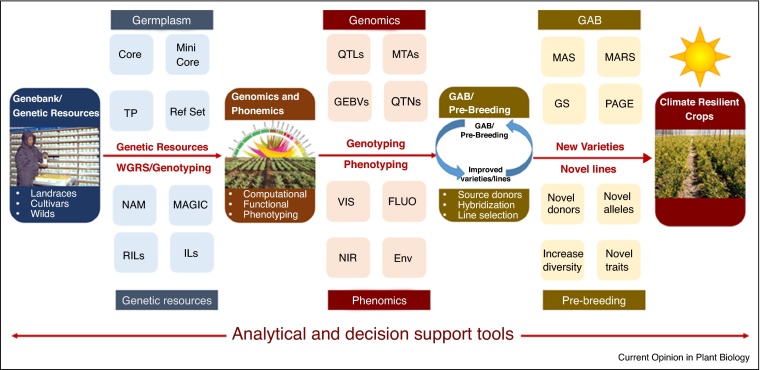
Integrated approach for development of climate-resilient crops. This figure presents an overview on use of germplasm/genetic resources together with genomics and phenomics approaches for identification of superior alleles/haplotypes and source donors for climate-change breeding related traits. Identified lines together with genomic information can be used in pre-breeding and genomics-assisted breeding (GAB) for faster delivery of climate-resilient crops. Although sequencing/genotyping of the entire genebank for a crop will be ideal, smaller subsets of germplasm such as reference set (Ref Set), core and mini core collections can be targeted initially. Similarly, specialized genetic resources/genetic stocks such as multi-parental (nested association mapping, NAM and multi-parent advanced generation intercross, MAGIC), bi-parental (recombinant inbred lines, RILs; introgression lines, ILs; F_2_) populations segregating for climate-change breeding related traits can be used. Training population (TP) can also be developed based on the specialized set of breeding lines for deployment of genomic selection (GS). These germplasm/genetic resources can be used for whole genome resequencing (WGRS) or high-density genotyping (e.g. genotyping-by-sequencing GBS, SNP-array based genotyping). High-throughput phenotyping of germplasm/genetic resources especially for climate-change breeding related traits can be undertaken using the visible light (VIS), the near-infrared (NIR) spectrum and fluorescence imaging (FLUO) in different environments (Env) by utilizing recent advances in sensors and imaging based phenotyping technologies. Analysis of these sequencing/genotyping data and phenotyping data using analytical and decision support tools (ADSTs) can provide quantitative trait loci (QTLs), marker trait associations (MTAs), quantitative trait nucleotides (QTNs), and genomic estimated breeding value (GEBVs). In addition, a catalogue of superior haplotypes and source donors for a given trait can also be identified. This information can be used in pre-breeding and GAB approaches. For instance, pre-breeding approaches by using novel donors can introduce novel alleles in the elite/cultivated genepool from the un-adapted germplasm (landraces, crop wild relatives) coming from harsh and extreme environmental conditions (simulating climate-change scenario). As a result, in addition to developing better pre-breeding lines, genetic diversity of the elite genepool can also be broadened. In the end, a number of GAB approaches such as marker assisted selection (MAS), marker-assisted recurrent selection (MARS) and GS can be deployed for integrating/accumulating superior alleles for climate-change breeding related traits. QTNs can be edited through genome editing approach called promotion of alleles through genome editing (PAGE). It is important to use ADSTs to analyze and make decision in every component of mining and integration of superior alleles in crop improvement programs. Integration of such approaches should accelerate development of climate-resilient cultivars with improved yield, enhanced resistance/tolerance to anticipated biotic and abiotic stresses and deliver higher genetics gains in farmer's fields especially in developing world.

In this review, we discuss strategies for identification of superior alleles/haplotypes from the genebanks, development and use of genotyping and phenotyping platforms and deployment of GAB approaches for pre-breeding and development of climate-change ready crops. We also present the challenges and opportunities toward integration of multidisciplinary research platforms/disciplines for developing climate change ready crop varieties (Figure 1).

## Identification of superior alleles/haplotypes

Approximately 7.4 million accessions are stored in 1700 seed banks globally, offering breeders a wealth of natural variation. Genebank passport data coupled with climatic data could be used as surrogates for abiotic stresses to identify genotypes that harbor important haplotypes which can then be integrated into breeding programs. To enhance the genetic gains through the incorporation of new sources of genetic variation, novel and superior genes need to be identified from crop wild relatives (CWR) and landraces available in genebanks [12, 13]. There are many examples where NGS based approaches are being successfully used to identify DNA polymorphisms associated with traits of interest. For instance, The 3000 Rice Genome [14^••^] and The 3000 Chickpea Genome Sequencing Initiative [15] offer opportunities to identify novel variations for a large number of genes through genotype-phenotypic associations. Re-sequencing of a large number of germplasm accessions not only provides information on the origin, domestication, and population structure [16•, 17••], but also identifies lines with deleterious mutations in the genomes that can eliminated to minimize the genetic load in the crop species [18]. NGS technologies together with precise phenotyping have been used for identification of marker trait associations in several crops, for example, rice [19^••^], soybean [20], pigeonpea [16^•^], foxtail millet [21] and pearl millet [17^••^]. Insights gained from these studies include new information on the genetic architecture of agriculturally important traits and the identification of valuable and sometimes novel alleles/haplotypes for morphological, agronomic, developmental and quality-related traits for enhancing genetic gains. In the context of developing climate change ready crops, it is essential to mine and incorporate superior alleles adapted to harsh and extreme environmental conditions by resequencing and phenotyping many germplasm accessions for the crop species.

Specialized mapping populations can be used to enhance the power and efficiency of genome wide association studies (GWAS). Nested association mapping (NAM) populations were first developed for maize as a way of taking advantage of both historical and recent recombination events [22]. This was important to minimize the density of markers required for GWAS and take the advantage of the high allelic richness, high mapping resolution, and high statistical power of association mapping. Multi-parent advanced generation inter-cross (MAGIC) populations [23, 24•] are another type of specialized mapping population, which are used to shuffle the genetic background by allowing several rounds of recombination in the genomes of diverse parental lines [25]. Both types of populations have been successfully developed and used to identify QTLs for a number of traits in diverse crop species for example, maize [26], wheat [27], rice [28] and cowpea [29]. Where robust donors are available for targeted traits, bi-parental mapping populations, that is, doubled haploid, recombinant inbred lines, introgression lines, and F_2_ populations are still useful in the identification of significant genomic region(s) for the trait of interest.

## Genetic analysis of climate change relevant traits

### High-throughput and cost-effective genotyping and phenotyping

Genotyping of all individual samples or selected recombinants of the targeted population either for trait mapping or product development is a critical step for identifying better alleles and/or superior lines for development of climate-resilient crops. The reliability, turn-around-time, ease of information retrieval, as well as the cost of a genotyping assay are critical to a breeder for making decisions about selection of individuals to advance to the next generation. Several genotyping platforms that leverage new technologies to discover and simultaneously genotype single nucleotide polymorphisms (SNPs) are currently available [30, 31]. Some of the most widely used sequencing-based genotyping approaches are genotyping-by-sequencing (GBS), restriction-site-associated DNA sequencing, double digest restriction associated DNA, skim-based genotyping by sequencing, repeat amplification sequencing, exome sequencing and whole genome re-sequencing (WGRS). Fixed SNP genotyping arrays may be preferred over NGS based technologies due to higher throughput at a lower cost per sample with minimum data analysis required. Rasheed and colleagues [32^•^] recently compiled the available crop breeding chips and genotyping platforms, which could be utilized in crop breeding programs.

Phenotyping, at present, is a significant operational bottleneck that limits the power and resolution of many genetic analyses. Robust, precise and high-throughput phenotyping systems are required for measuring a full suite of genetic factors that contribute to quantitative phenotypic variation across cells, organs, and tissues, developmental stages, years, environments, species and research programs [33]. A fundamental advance in high-throughput phenotyping platforms is the capability to non-destructively capture plant traits [34]. Recent advances in sensors for imaging plants, ranging from remote sensing including spectroradiometry, Light Detection and Ranging (LiDAR), visible to far-infrared, hyperspectral, thermal, fluorescence, and 3D laser scanning to trichromatic imaging in conjunction with advanced autonomous vehicles, have indeed opened up the possibility of high-throughput phenotyping [35, 36]. Autonomous platforms such as unmanned aerial vehicles and ground robots equipped with multiple sensors can take pictures in near real-time of the entire experimental plot several times per day, or over the entire season from germination to maturity, resulting in massive amounts of data for analysis and storage [37]. Use of such phenotyping platforms for measuring traits on germplasm collections, adapted to harsh conditions, in simulated climate change conditions will help to identify the genes and lines that cope with future climatic conditions.

### Rapid trait mapping

Advances in genomics have led to the development of NGS based trait mapping approaches, which have speed up trait mapping programs from a few years to just a few months. For instance, NGS technologies have enabled modification and improvement of time-consuming bulked segregant analysis [38] into rapid and whole-genome sequencing based high-resolution trait mapping [39•, 40]. The availability of draft genome sequences for a number of crop species and reduction in sequencing costs have also made it possible to resequence hundreds to thousands of individuals of a genetic population. As a result, the genetics community was able to deploy WGRS or low coverage sequencing of the entire population or bulks of the extremes. GBS and WGRS of entire mapping populations or extreme pools provide large-scale genome-wide SNPs for conducting high-resolution trait mapping, and several examples have been reported in many crop species [41]. On the other hand, bulk or pool-based sequencing has become popular for rapid trait mapping in recent years [42, 43, 44, 45].

## Next generation breeding approaches

### Pre-breeding for capturing novel alleles

Pre-breeding is required to identify and transfer desirable traits and genes from un-adapted materials to intermediate materials [46]. The breeders can use these intermediate materials further in producing new varieties. It is a first essential step in the ‘linking genetic variability to utilization’ use of diversity arising from CWR and other un-adapted materials. A collaboration between the germplasm curator and the plant breeder is essential for bringing new traits from these collections into newly bred varieties. The decision for pre-breeding is based on the anticipated effectiveness and efficacy of transferring the target traits into cultivars and source of the desired gene(s). Pre-breeding can be useful in: first, broadening the genetic base, second, identifying and characterizing climate change relevant traits in exotic materials, third, identification and introgression of genes from wild species or unadapted material into breeding populations, and fourth, identification and transfer of novel genes from unrelated species using genetic transformation techniques.

It is important to note that working with CWR in pre-breeding program is a challenging task especially due to the crossing barrier and introduction of linkage drag from CWR in the elite genepool. Recently, Demepewolf and colleagues [47] provided strategies to deal such challenges after undertaking extensive literature search and in-depth interviews with the experts. For instance, embryo rescue approach can be useful to handle interspecific crossability. Genomics technologies are very helpful for identification of the markers linked with useful segments in CWR that can be used for introgression and minimizing linkage drag. Furthermore, genome editing technologies, once established in a given species, can also be used to repair deleterious effect CWR allele(s) and convert un-adapted material into superior lines [48].

Access and benefit-sharing agreements (ABS) arising from the use of genetic resources (including CWR) should be shared in a fair and equitable manner between different stakeholders for successfully running the pre-breeding program. Additionally, access to pre-breeding materials, open access data, and *in situ* and *ex situ* agricultural diversity conservation of pre-breeding materials are also some important components of pre-breeding. Though there are several success stories for accessing superior alleles through pre-breeding, it is important to have long-term and adequate funding for pre-breeding to have accelerate access of superior alleles related to climate change related traits.

### Genomics-assisted breeding for rapid development of superior lines

A number of molecular breeding approaches have been used to introgress genomic regions into elite lines [49]. Marker-assisted selection is useful to introgress a few loci (<10 loci) for improving elite varieties. It has been widely used in a number of crops to incorporate desired traits into elite cultivars through marker-assisted backcrossing (MABC). Nowadays there is an emphasis on early generation selection by using the forward breeding (FB) approach [15]. In this approach, a set of diagnostic markers for must-have traits is used to screen early generations and a subset of lines is advanced to the next generation. This approach is useful for enhancing the selection intensity to accelerate the genetic gains. Marker-assisted recurrent selection may be appropriate for more complex traits controlled by up to 40 loci. This approach can be used to develop superior lines with an optimum combination of superior alleles through repeated inter-crossing [9^••^].

Genomic selection (GS) has been used extensively in animal breeding programs and in the last 10 years has become popular for improving the rate of genetic gain in crop breeding. GS employs genomic estimated breeding values (GEBVs) of lines in a segregating population, which are calculated based on the genotypic and phenotypic dataset of a ‘*training population*’ [50]. This approach is advantageous for quantitative traits and increases selection efficiency by shortening breeding cycles. GS has been applied in many crops, and recently it has been reviewed by Crossa *et al.* [51^•^]. Speed breeding coupled with GS (SpeedGS) has been promoted for rapid development of climate-resilient crops [11^••^].

Genome editing (GE) is a method that enables specific nucleotides in the genome of an individual to be changed. GE seems to be one of the promising approaches that could be conveniently exploited to generate homozygous mutants for multiple target genes in a single generation. This implies that new varieties could be developed much faster than usual traditional or even molecular breeding methods. In addition, GE technology is also very useful for generating targeted variations, thereby broadening the allele pool for precision breeding [48, 52]. Most importantly, the resultant product of genome editing, as per the scientific community, is not a genetically modified organism (GMO) [53]. Therefore, the GE approach, although superior and much more precise than genetic engineering, is likely not to face regulatory and public acceptance [54].

A large number of genes for targeted traits in many crop species have been cloned, and underlying quantitative trait nucleotides (QTN) with significant effects have been identified. Genes with defined QTNs that cause a sizeable phenotypic effect can be modified by GE. It is also possible to combine GE and GS and the strategy has been referred to as the promotion of alleles by genome editing (PAGE) [55]. In the near future, alleles that have a positive effect in the targeted phenotype can be transferred through genomics-based approaches (MABC and FB) and alleles that have an adverse effect in the targeted phenotype can be corrected through PAGE/genome editing.

Once superior breeding lines are developed, evaluation of these lines in the target population of environments (TPEs) is the crucial component for selecting the lines that will perform better in changing climate conditions. In fact, breeding programs now need to place greater emphasis on increasing the TPEs for evaluation than on development of more lines and phenotyping them at a limited number of locations.

## Conclusions and prospects

While working on germplasm collections including CWR, the genetics community can map climate change relevant traits with the help of high-throughput genotyping and phenotyping platforms in a faster and more cost-effective manner. Next generation breeding approaches including GS and GE can use the new germplasm and technological advances to develop climate change ready lines. Public crop improvement programs have pioneered the development of new technologies and breeding methods. To capture these opportunities [56^••^] more co-ordination is needed so that these advances can be translated into delivering higher genetic gains in farmers’ fields, particularly in the developing world. Therefore, to mitigate the current challenges we have to deploy a unified strategy to make a more significant impact through integrating different disciplines and to increase selection intensity, selection accuracy, heritability and next generation breeding approaches to develop climate change ready crops in a cost-effective and rapid manner. To make the unified strategy workable, crop improvement programs (with multi-disciplinary scientists and not just breeders) need to set the priorities and take the advice from their stakeholders to develop a product profile. Team members need to work and complete their part in the value chain. The new lines with higher yield and adaptation to extreme conditions should be evaluated in the TPEs so that climate resilient crop varieties are developed quickly. Appropriate agronomical practices together with GIS can be useful for improving adoption of superior lines, for realizing enhanced yield in farmers’ fields and for providing more income to farmers. It is also essential to connect with the farming community, state governments and non-government organizations to support equitable ownership of resources and benefit sharing [57] so that the full potential of genetic resources can be fully realized for benefitting small holder farmers in developing world.

## Conflict of interest

The author(s) declare that they have no competing interests.

## References and recommended reading

Papers of particular interest, published within the period of review, have been highlighted as:

• of special interest
•• of outstanding interest
